# Measuring Meiotic Crossovers via Multi-Locus Genotyping of Single Pollen Grains in Barley

**DOI:** 10.1371/journal.pone.0137677

**Published:** 2015-09-10

**Authors:** Steven Dreissig, Jörg Fuchs, Petr Cápal, Nicola Kettles, Ed Byrne, Andreas Houben

**Affiliations:** 1 Leibniz Institute of Plant Genetics and Crop Plant Research (IPK) Gatersleben, Corrensstrasse 3, 06466 Stadt Seeland, Germany; 2 Institute of Experimental Botany, Centre of the Region Haná for Biotechnological and Agricultural Research, Šlechtitelů 31, Olomouc CZ-78371, Czech Republic; 3 KWS-UK Ltd, 56 Church Street, Thriplow, Hertfordshire, SG8 7RE, United Kingdom; National Cancer Institute, UNITED STATES

## Abstract

The detection of meiotic crossovers in crop plants currently relies on scoring DNA markers in a segregating population or cytological visualization. We investigated the feasibility of using flow-sorted haploid nuclei, Phi29 DNA polymerase-based whole-genome-amplification (WGA) and multi-locus KASP-genotyping to measure meiotic crossovers in individual barley pollen grains. To demonstrate the proof of concept, we used 24 gene-based physically mapped single nucleotide polymorphisms to genotype the WGA products of 50 single pollen nuclei. The number of crossovers per chromosome, recombination frequencies along chromosome 3H and segregation distortion were analysed and compared to a doubled haploid (DH) population of the same genotype. The number of crossovers and chromosome wide recombination frequencies show that this approach is able to produce results that resemble those obtained from other methods in a biologically meaningful way. Only the segregation distortion was found to be lower in the pollen population than in DH plants.

## Introduction

Meiotic recombination is the primary mechanism of generating novel allelic combinations and introducing genetic diversity. In barley (*Hordeum vulgare* L.), as well as in many other crops, recombination frequencies are elevated in distal gene-rich chromosomal regions. Nevertheless, 24.7% of the total barley gene content is located in low recombining regions [1] representing an untapped resource which is unavailable for plant breeding [2]. Hence, strategies to modulate the recombination frequency along chromosomes are needed. The ability to induce an increase in meiotic recombination is so far limited to the model species *Arabidopsis thaliana* via a mutation of the FANCM helicase [3]. In barley, Higgins et al. [4] demonstrated a shift of meiotic crossovers towards interstitial and proximal regions at higher temperatures during meiosis.

There are different ways of monitoring meiotic crossovers in plants. They can be identified using molecular markers in a segregating population [5], or alternatively the frequency and distribution of crossovers can be visualized by cytological means like analysis of pairing configurations [6] or immunolabeling of proteins involved in meiotic recombination such as the barley MutL homologue (HvMLH3) [7]. One limitation of using microscopy-based methods is that the sites of recombination events can only be resolved at the chromosomal level. Another limitation is an uncertainty about perfect agreement between protein localization and crossover. Other tools for efficient determination of recombination events such as the tetrad analysis based on the quartet (qrt) mutation are currently only available for *Arabidopsis thaliana* [8].

In human and livestock genetics, recombination analysis using meiotic gametophytes was developed more than 20 years ago for the high-resolution mapping of recombination sites. In plants, the idea of analysing pollen grains has already brought forward a number of studies. Petersen et al. [9] extracted DNA from single barley and rye pollen grains for PCR amplification and subsequent sequencing of high and single copy genes. Chen et al. [10] developed a method using pollen grains of several plant species for molecular analysis utilizing randomly amplified polymorphic DNA and simple sequence repeat markers. The introduction of whole-genome-amplification (WGA) methods, such as primer extension pre-amplification, enabled Aziz and Sauve [11] to further increase the amount of information gained from single pollen grains. However, other WGA methods based on isothermal amplification via the Phi29 DNA polymerase hold the potential to enable the analysis of hundreds to thousands of markers in a single cell [12].

In the current study, we describe a strategy to perform a parallel analysis of individual haploid nuclei derived from pollen grains by utilizing fluorescence activated cell sorting (FACS) coupled with Phi29 DNA polymerase-based whole-genome-amplification (WGA) and multi-locus KASP genotyping. The meiotic crossover measurements were compared to data obtained by different methods in comparable genetic environments.

## Materials and Methods

### Plant material and isolation of pollen nuclei and genomic DNA

Pollen grains were collected from an F_1_ plant of the barley cultivars Morex x Barke (*Hordeum vulgare* L.). Mature anthers of 20 flowers were collected in a 1.5 ml Eppendorf tube using forceps. Afterwards, 300 μl of ddH_2_O were added and the suspension was vortexed for approximately 30 sec. The suspension was shaken at 1500 rpm for 10 min at room temperature to release all pollen grains. Afterwards, the pollen suspension was centrifuged for 5 min at 13,000 rpm and all empty anthers were manually removed using forceps. After centrifugation for 5 min at 13,000 rpm the supernatant was removed and the pollen pellet was resuspended in 100 μl Galbraith buffer (45 mM MgCl_2_, 30 mM sodium citrate, 20 mM MOPS, 0.1% Triton-X100, pH to 7.0; [13]) and transferred into a 2.0 ml Eppendorf tube containing two metallic beads of 6 mm in diameter (Intec GmbH) as described in [14]. The pollen-bead mixture was centrifuged for 5 min at 13,000 rpm prior to homogenization at 30 Hz for 40 seconds using a MM 400 ball mill (Retsch). After homogenization, another 500 μl Galbraith buffer were added and the suspension was filtered through a 30 μm filter (Sysmex-Partec). For the purpose of providing genomic DNA for marker testing, genomic DNA was extracted from leaf tissue using the DNeasy Plant Mini kit (Qiagen) and measured using nanodrop (Peqlab).

### FACS-based purification of single haploid nuclei and whole-genome-amplification

The nuclei suspension was stained with 4',6-diamidino-2-phenylindole (DAPI; 1.5 μg/ml) and single 1C nuclei were sorted using a BD FACSAria IIu (BD Biosciences) flow-sorter into individual wells of a 384-microwell plate containing 2 μl lysis solution (0.5 μl lysis buffer composed of 400 mM KOH, 100 mM DTT, 10 mM EDTA; [15], 0.5 μl ddH_2_O, 1 μl sample buffer (Genomiphi V2, GE Healthcare)) for whole-genome-amplification. Note, in contrast to the manufacturer’s protocol the sample buffer containing random primers for whole-genome-amplification was added to lysis solution. Whole-genome-amplification was carried out using the Genomiphi V2 kit (GE Healthcare) according to the manufacturer’s protocol with the following modifications: Nuclei lysis and DNA denaturation was conducted by incubation at 65°C for 3 min in 2 μl lysis solution. The lysis solution was neutralized by adding 0.5 μl neutralisation buffer (666 mM Tris-HCl, 250 mM HCl; [15]). Afterwards, a master mix composed of 3.5 μl sample buffer, 4.5 μl reaction buffer and 0.5 μl enzyme mix (all Genomiphi V2, GE Healthcare) per reaction was added and samples were incubated at 30°C for 8 hours followed by inactivation of the enzyme at 65°C for 10 minutes. Subsequently, each sample was diluted with 500 μl ddH_2_O. The DNA concentration of the WGA products of single pollen nuclei was measured by fluorometric quantitation (Qubit, Life Technologies).

Each sample was subjected to a PCR using primers for the *Ty3/gypsy*-like retroelement *cereba* in order to validate the successful sorting of pollen nuclei into the microwells. The reaction volume of the *cereba* amplification was 10 μl containing 5 μl WGA product, 1x PCR buffer (Qiagen), 0.2 mM dNTPs (Bioline), 1x Q-solution (Qiagen), 0.6 μM of each primer and 0.02 units Taq DNA polymerase (Qiagen). The following thermal cycling conditions were used: DNA polymerase activation: 3 min at 95°C; denaturation: 30 sec at 95°C; annealing: 30 sec at 60°C; extension: 30 sec at 72°C; final extension: 10 min at 72°C; 30 cycles in total. The *cereba*-positive samples were further analysed with 8 chromosome 3H-specific primers to quantify the efficiency of the whole-genome-amplification (S1 Table). These primer pairs were targeting single copy sequences to test if the WGA was able to amplify unique sequences. The reaction volume of the 3H-specific amplification was 10 μl containing 5 μl WGA product, 1x PCR buffer (Qiagen), 0.2 mM dNTPs (Bioline), 1x Q-solution, 0.3 μM of each primer and 0.02 units Taq DNA polymerase (Qiagen). The following thermal cycling conditions were used: DNA polymerase activation: 3 min at 95°C; denaturation: 30 sec at 95°C; annealing: 30 sec at 65°C, reduced by 1°C for 9 cycles; extension: 30 sec at 72°C; 25 cycles at final annealing temperature.

### KASP-genotyping

A set of 24 chromosome 3H-specific single nucleotide polymorphisms (SNPs) [16] based on the current barley genome sequence assembly [17] was chosen and converted into KASP markers (LGC Genomics, S2 Table). Thermal cycling conditions were adopted from Mirouze et al. [18] and end-point signals were read out on a BioRad iQ5 cycler at 30°C. Genomic DNA from cv. Morex, cv. Barke and Morex x Barke F_1_ plants were genotyped in parallel. Additionally, replicate single pollen nuclei of the cultivar Morex were subjected to whole-genome-amplification and subsequent genotyping to act as a positive control against amplification errors. This was done in order to test the possibility of false allele calling due to inaccurate WGA or contamination. Allele calling was done manually by plotting relative fluorescence values of FAM and HEX against each other. Heterozygous signals were discarded as genotyping errors since we expect haploid nuclei to only provide homozygous signals.

### Analysis of segregation distortion loci and crossovers

To test for segregation distortion we conducted a χ²-test assuming an expected segregation ratio of 1:1 for each marker. Segregation distortion loci (SDL) were identified by significant deviation from the expected ratio of 1:1 (P < 0.05). Crossovers were detected by visualizing our marker data using flapjack [19] by identifying allele calls for which there was a switch from allele A (Morex) to allele B (Barke) and vice versa. The physical position of each marker on barley chromosome 3H was derived from the barley genome sequence assembly [17] thus enabling us to count crossovers without constructing a linkage map. The recombination frequency between two adjacent marker pairs was measured as the proportion of crossovers to no-crossovers. Marker pairs within a sample involving missing data points were omitted from the analysis. The number of crossovers was normalized according to the following calculation:

$$\mathrm{CO ratio}=\frac{n\;(\mathrm{CO})}{n\;(\mathrm{no}-\mathrm{CO})+n\;(\mathrm{CO})}$$ (1)

where n(CO) is the number of crossovers and n(no-CO) is the number of no-crossovers.

Similarly, the number of no-crossovers was normalized according to the following calculation:

$$\mathrm{no}-\mathrm{CO ratio}=\frac{n\;(\mathrm{no}-\mathrm{CO})}{n\;(\mathrm{no}-\mathrm{CO})+n\;(\mathrm{CO})}$$ (2)

The ratio of (1) to (2) enabled us to calculate recombination frequencies for each marker pair in a comparable manner. To compare our data to the Morex x Barke DH population data, we used raw genotyping-by-sequencing data (wheat.pw.usda.gov, S3 Table) and extracted the physical map position for each marker [17]. Segregation distortion and crossover analysis of the Morex x Barke DH population was performed as described above. A two-tailed unpaired Student’s t-test was performed to compare the average number of crossovers in both populations and the distribution of the number of crossovers was compared using a χ²-test assuming the Morex x Barke DH crossover data as expected values.

## Results and Discussion

### Whole-genome-amplification of single haploid nuclei

In order to develop a strategy for the high-throughput analysis of meiotic recombination events in barley pollen, we first needed to prove the successful extraction, amplification and genotyping of single pollen DNA. To overcome problems associated with the rigid cell wall of pollen grains described by Chen et al. [10], we selected a novel approach to isolate haploid nuclei suitable for flow-sorting.

**Fig 1:**
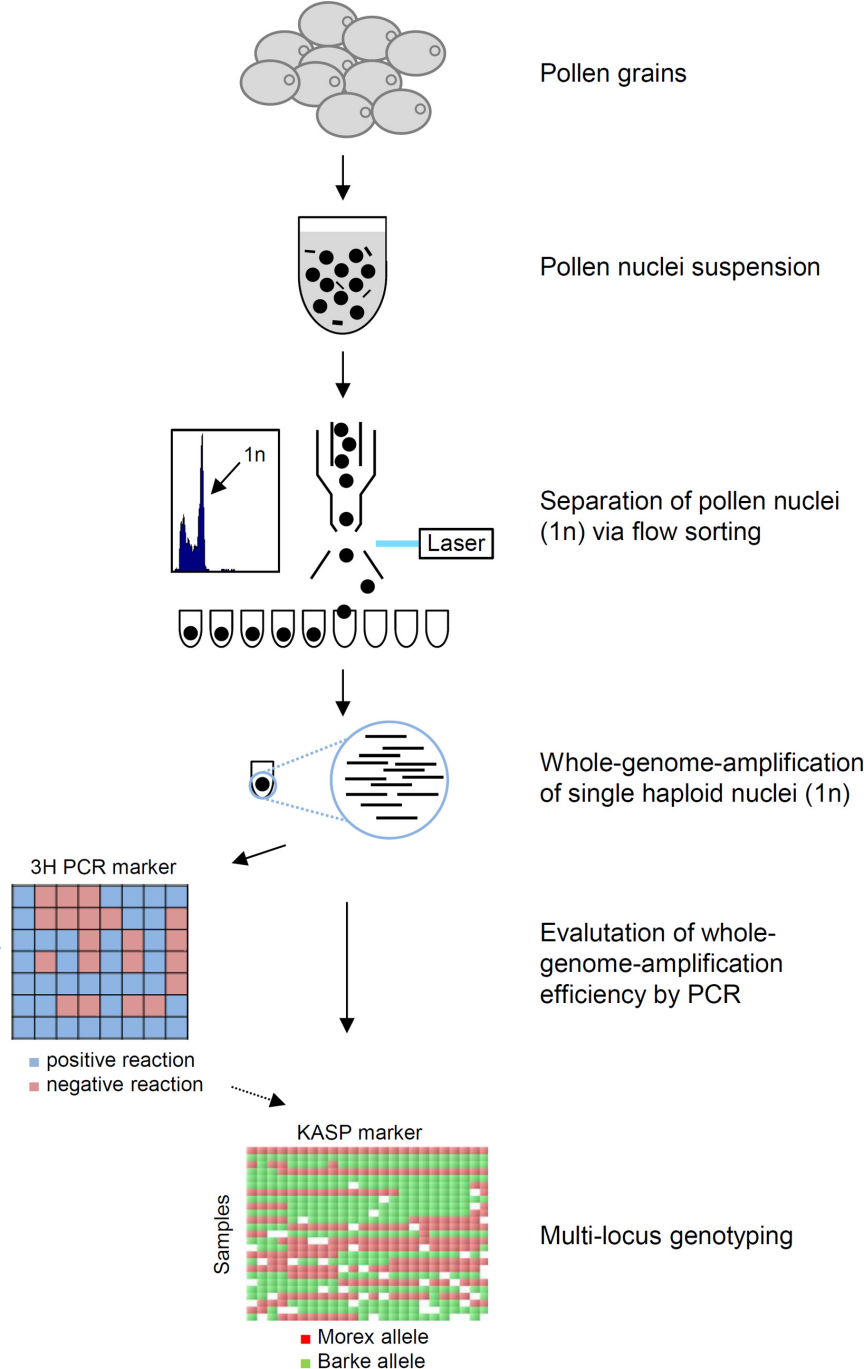
Scheme of experimental workflow developed in the current study. Haploid nuclei were extracted from pollen grains, separated via flow-sorting and individually subjected to whole-genome-amplification (WGA). High quality samples, evaluated by PCR with chromosome 3H-specific primers, were genotyped using 25 KASP markers to measure crossover frequency and distribution.

**Fig 2:**
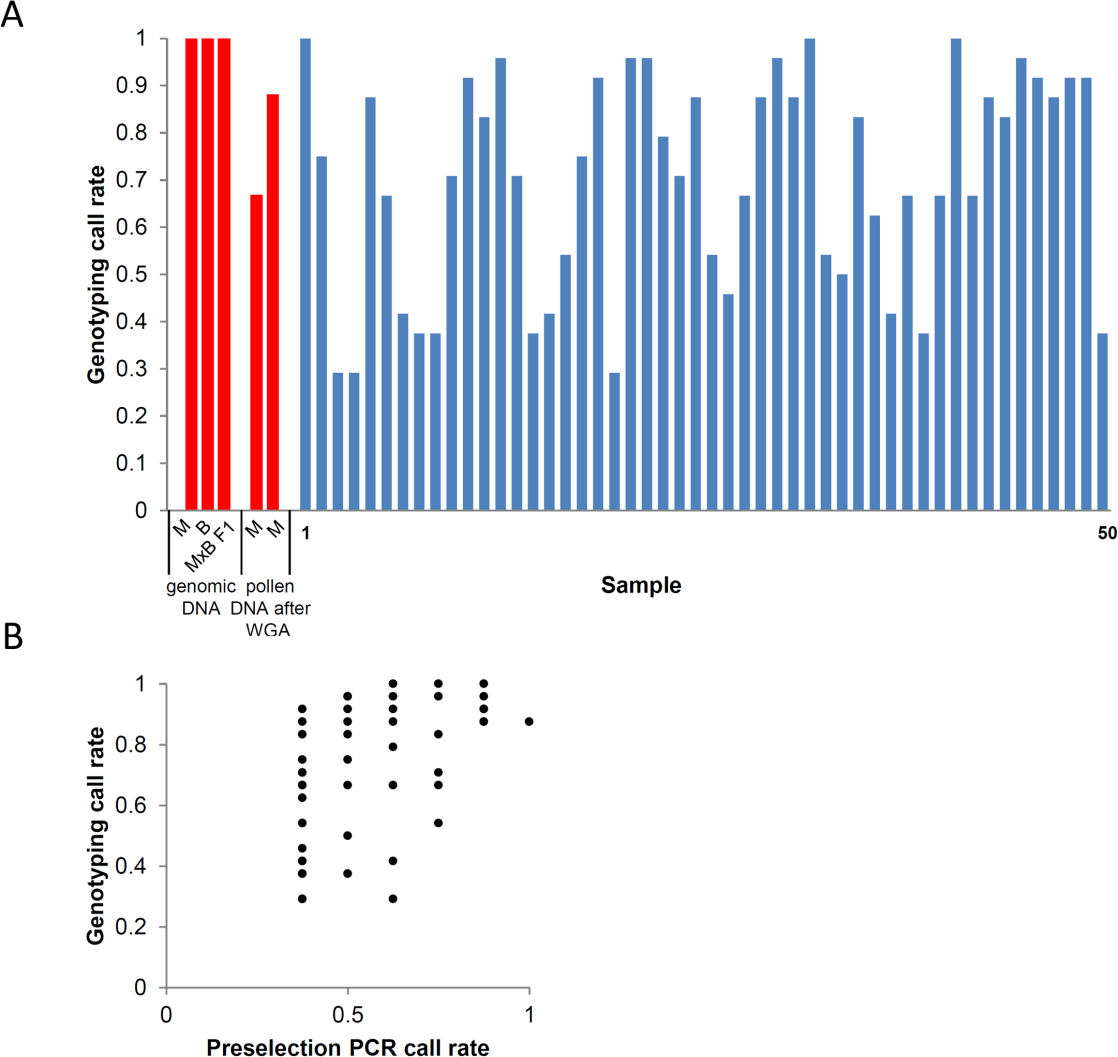
KASP genotyping performance. **(A)** Genotyping call rate of the positive controls across 24 KASP markers is indicated in red. Genomic DNA from Morex, Barke and Morex x Barke F1 plants was used. Two individual nuclei derived from Morex pollen grains were used to test for false allele calling due to whole-genome-amplification. The selected 50 Morex x Barke pollen nuclei samples are indicated in blue showing an average genotyping call rate of 71%. (**B)** Correlation between KASP genotyping call rate and preselection PCR call rate. r = 0.51, r² = 0.26.

Isolated haploid nuclei from pollen grains were individually sorted via a FACS-based approach into individual wells of a 384-microwell plate (Fig 1). After nuclei lysis and DNA denaturation, whole-genome-amplification was performed using Phi29 DNA polymerase. Each reaction yielded 1 to 3 μg of DNA consisting of barley-specific products and likely also unspecific products as expected from whole-genome-amplification via Phi29 DNA polymerase [20]. The products of 192 single-nuclei whole genome amplification (WGA) reactions were analysed by PCR for the presence of the barley high copy *Ty3/gypsy*-like retroelement *cereba* to confirm the successful sorting of nuclei into the individual microwells. From a total of 192 samples, 168 contained PCR amplified barley DNA giving an accuracy of our FACS approach of 87.5%. To preselect samples for further genotyping, we used PCR to amplify eight 3H-specific single copy sequences located across both arms of chromosome 3H. Out of the 168 single-nuclei amplifications positively tested for *cereba*, we selected 50 samples which showed successful amplifications of at least 3 single copy sequences from both arms for further genotyping (S1 Fig). To measure meiotic crossovers on chromosome 3H, we selected 24 KASP markers. The suitability of the 24 selected chromosome 3H-specific KASP markers was confirmed using genomic DNA isolated from leaves of both genotypes. Next, the same set of markers was used to genotype WGA-amplified DNA derived from individual haploid nuclei. The selected 50 nuclei samples revealed an average marker call rate of 70%, indicating that the majority of samples were effectively amplified (Fig 2A). Considering preselection via PCR, we found a weak but yet positive correlation between final genotyping call rate and preselection PCR call rate (r = 0.51, r² = 0.26, Fig 2B) which indicates the advantage of preselecting samples after WGA before conducting downstream analyses. Only three samples resulted in products with less than 35% of the markers, probably due to inefficient amplification of the Phi29 polymerase-based whole-genome-amplification system, as described previously [12, 20]. 98% of the positive KASP reactions (1226 out of 1250) showed clear homozygous signals in agreement to the positive-control. 24 heterozygous calls (1.92%) were observed. These were randomly distributed across all samples and markers and therefore unlikely to be caused by an erroneous sorting of two nuclei into one microwell, so they were discarded as genotyping errors. Furthermore, no false allele calling, e.g. Barke allele instead of Morex, due to WGA errors was found in the positive controls using haploid nuclei of Morex. We conclude that multi-locus KASP-based genotyping on WGA-amplified DNA derived from single haploid nuclei is feasible.

### Monitoring meiotic recombination by genotyping single pollen grains

**Fig 3:**
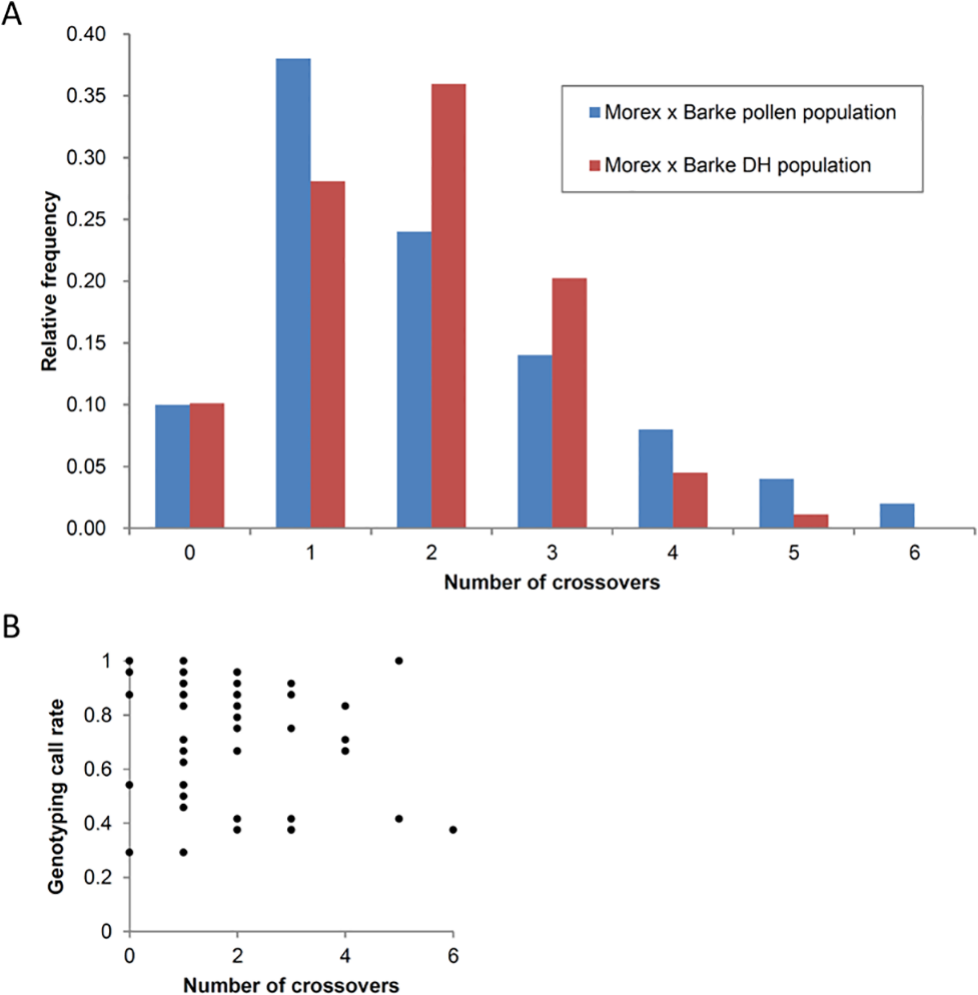
Comparison of the distribution of the number of crossovers. **(A)** The relative frequency of the total number of crossovers per chromosome 3H grouped into classes ranging from 0 to 6 of the Morex x Barke pollen population (blue) in comparison to the Morex x Barke DH population data (red) [17]. (**B)** Correlation between KASP genotyping call rate and the number of crossovers found for each sample (r = -0.16, r² = 0.03).

**Fig 4:**
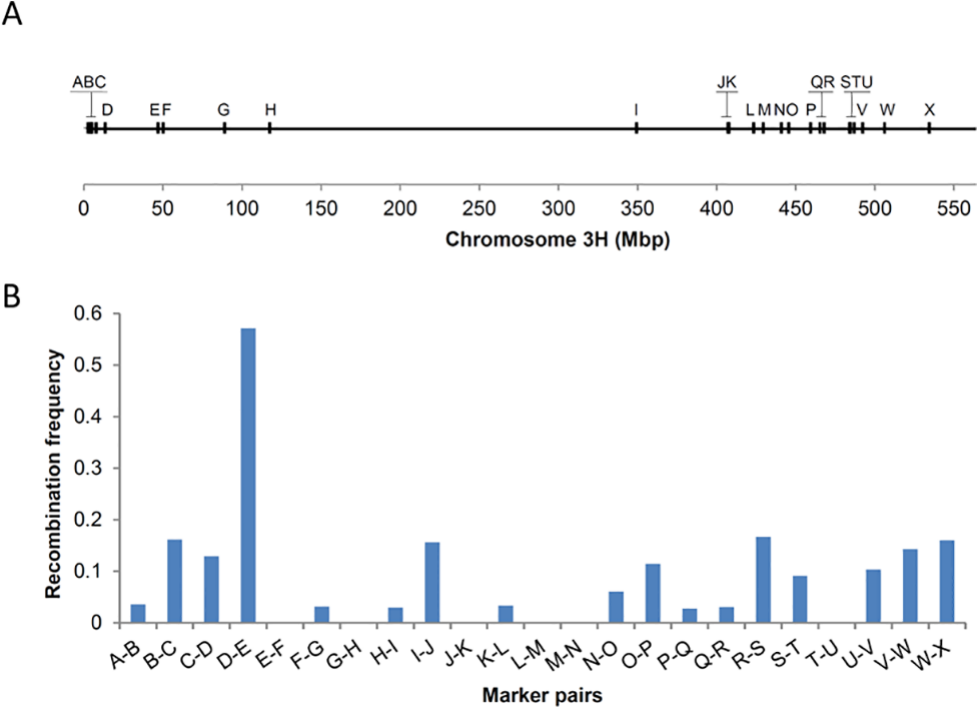
Recombination frequency along chromosome 3H determined by pollen genotyping. **(A)** KASP marker positions (A to X) are shown as vertical bars along chromosome 3H. The physical length of the chromosome (Mbp) is shown on the x-axis. **(B)** The recombination frequency along chromosome 3H of a given physical interval measured as the proportion of crossovers to no-crossovers for each marker pair. A distal bias is shown by higher recombination frequencies towards the chromosome ends and low recombination frequencies between markers H and I.

Meiotic crossovers along chromosome 3H were measured with a mean inter-marker distance of 14.35 and 12.32 mega base-pairs (Mbp) for the short and long arm of chromosome 3H, respectively [17]. The average number of crossovers for chromosome 3H in our pollen population was 1.92, while the corresponding number for a corresponding DH population was 1.84. These two values are not significantly different (*P* = 0.72). Looking at the distribution of the total number of crossovers, we did not find a significantly different pattern for both pollen and DH population (Fig 3A) indicating the reliability of our approach (χ²-test, *P* = 0.99). Although one individual nucleus showing 6 crossovers on chromosome 3H was found to have a low genotyping call rate of 0.38, there was no significant correlation between the number of crossovers and genotyping call rate (r = -0.16, r² = 0.03, Fig 3B). We further determined recombination frequencies along chromosome 3H by counting the number of crossovers in neighbouring marker intervals (Fig 4). A typical pattern of elevated recombination frequencies towards the distal regions of the chromosome was found which is in agreement with previous reports for barley based on molecular marker data [1, 21] and cytological visualizations of crossovers [7].

To assess the extent of segregation distortion in pollen grains, we investigated the number of loci showing segregation distortion in our pollen population and compared it to equivalent data derived from a doubled haploid (DH) population of the same genotype. In our pollen population, 24 loci on chromosome 3H were scored for presence or absence of each allele. Segregation distortion was found for 8.3% (2 of 24) of the markers (S3 Table). This proportion appears to be lower compared to the Morex x Barke DH population which showed 25.7% (25 of 97) of all loci on chromosome 3H having distorted segregation ratios (S3 Table). However, the difference in sample size, which is 50 pollen grains compared to 89 DH individuals, allows only major effects to be detected. This difference might be explained by selection against particular genotype combinations during anther culture of the DH lines or by absence of selection in pollen grains for pollination and fertilization success. However, this tendency is in agreement with Sayed et al. [22] who compared segregation distortion of a barley DH population versus an F_2_ population, finding a difference of 44.2% versus 16.3%, respectively.

We conclude that it is feasible to genotype single pollen grains using our amplification approach combined with KASP. It offers the opportunity to efficiently monitor meiotic recombination in individual pollen nuclei and avoids the necessity to generate segregating populations. Due to the high amount of DNA obtained from a single haploid nucleus via WGA, we suggest that our approach might be used for genome wide analyses. This will be particularly useful in plant breeding to monitor the recombination landscape of any genotype of interest.

## Supporting Information

S1 Fig Distribution of the number of positive PCR markers to assess WGA efficiency. (TIF)

S2 Fig Distribution of KASP marker call rate and frequency of double crossover. The marker call rate of each KASP marker (A to X) is shown (blue) as well as the frequency of presumptive double crossover (red), e.g. a crossover on both sides of a given marker.
(TIF)

S1 Table List of primers used to evaluate WGA performance. (DOCX)

S2 Table Gene-based KASP markers. (DOCX)

S3 Table Genotypic data and segregation statistics of the pollen population and DH population. Sample number is shown in the first column and the first row indicates the marker number. Genotypic values are shown as A for Morex and B for Barke. Missing values are indicated by a minus.
(XLSX)

S4 Table Supplementary information to Figs 2, 3, 4 and S5, S6 Figs. Actual data referring to Figs 2, 3, 4 and S5, S6 Figs are summarized in separate tables.
(XLSX)

## References

[1] Baker K, Bayer M, Cook N, Dreissig S, Dhillon T, Russell J, et al The low-recombining pericentromeric region of barley restricts gene diversity and evolution but not gene expression. Plant J. 2014;79(6):981–92. 10.1111/tpj.12600 . 24947331 PMC4309411

[2] Tondelli A, Xu X, Moragues M, Sharma R, Schnaithmann F, Ingvardsen C, et al Structural and Temporal Variation in Genetic Diversity of European Spring Two-Row Barley Cultivars and Association Mapping of Quantitative Traits. Plant Genome-Us. 2013;6(2). 10.3835/plantgenome2013.03.0007 .

[3] Crismani W, Girard C, Froger N, Pradillo M, Santos JL, Chelysheva L, et al FANCM limits meiotic crossovers. Science. 2012;336(6088):1588–90. 10.1126/science.1220381 . 22723424

[4] Higgins JD, Perry RM, Barakate A, Ramsay L, Waugh R, Halpin C, et al Spatiotemporal Asymmetry of the Meiotic Program Underlies the Predominantly Distal Distribution of Meiotic Crossovers in Barley. Plant Cell. 2012;24(10):4096–109. 10.1105/tpc.112.102483 . 23104831 PMC3517238

[5] Salome PA, Bomblies K, Fitz J, Laitinen RA, Warthmann N, Yant L, et al The recombination landscape in Arabidopsis thaliana F2 populations. Heredity. 2012;108(4):447–55. 10.1038/hdy.2011.95 22072068 PMC3313057

[6] Sybenga J. Quantitative Analysis of Chromosome Pairing and Chiasma Formation Based on Relative Frequencies of M I Configurations .4. Interchange Heterozygotes. Genetica. 1966;37(2):199–&. 10.1007/Bf01547131 . 5883031

[7] Phillips D, Wnetrzak J, Nibau C, Barakate A, Ramsay L, Wright F, et al Quantitative high resolution mapping of HvMLH3 foci in barley pachytene nuclei reveals a strong distal bias and weak interference. J Exp Bot. 2013;64(8):2139–54. 10.1093/jxb/ert079 23554258 PMC3654414

[8] Copenhaver GP, Keith KC, Preuss D. Tetrad analysis in higher plants. A budding technology. Plant Physiol. 2000;124(1):7–16. . 10982416 10.1104/pp.124.1.7 PMC1539273

[9] Petersen G, Johansen B, Seberg O. PCR and sequencing from a single pollen grain. Plant Mol Biol. 1996;31:189–91. 8704154 10.1007/BF00020620

[10] Chen PH, Pan YB, Chen RK. High-throughput procedure for single pollen grain collection and polymerase chain reaction in plants. J Integr Plant Biol. 2008;50(3):375–83. 10.1111/j.1744-7909.2007.00624.x . 18713371

[11] Aziz AN, Sauve RJ. Genetic mapping of Echinacea purpurea via individual pollen DNA fingerprinting. Mol Breeding. 2008;21(2):227–32. 10.1007/s11032-007-9123-9 .

[12] Wang JB, Fan HC, Behr B, Quake SR. Genome-wide Single-Cell Analysis of Recombination Activity and De Novo Mutation Rates in Human Sperm. Cell. 2012;150(2):402–12. 10.1016/j.cell.2012.06.030 . 22817899 PMC3525523

[13] Galbraith DW, Harkins KR, Maddox JM, Ayres NM, Sharma DP, Firoozabady E. Rapid flow cytometric analysis of the cell cycle in intact plant tissues. Science. 1983;220(4601):1049–51. 10.1126/science.220.4601.1049 . 17754551

[14] De Storme N, Geelen D. The Arabidopsis mutant jason produces unreduced first division restitution male gametes through a parallel/fused spindle mechanism in meiosis II. Plant Physiol. 2011;155(3):1403–15. 10.1104/pp.110.170415 21257792 PMC3046594

[15] Gole J, Gore A, Richards A, Chiu YJ, Fung HL, Bushman D, et al Massively parallel polymerase cloning and genome sequencing of single cells using nanoliter microwells. Nat Biotechnol. 2013;31(12):1126–32. 10.1038/nbt.2720 24213699 PMC3875318

[16] Comadran J, Kilian B, Russell J, Ramsay L, Stein N, Ganal M, et al Natural variation in a homolog of Antirrhinum CENTRORADIALIS contributed to spring growth habit and environmental adaptation in cultivated barley. Nature genetics. 2012;44(12):1388–92. 10.1038/ng.2447 . 23160098

[17] International Barley Genome Sequencing Consortium (IBGSC). A physical, genetic and functional sequence assembly of the barley genome. Nature. 2012;491(7426):711–+. 10.1038/Nature11543 . 23075845

[18] Mirouze M, Lieberman-Lazarovich M, Aversano R, Bucher E, Nicolet J, Reinders J, et al Loss of DNA methylation affects the recombination landscape in Arabidopsis. Proc Natl Acad Sci U S A. 2012;109(15):5880–5. 10.1073/pnas.1120841109 22451936 PMC3326504

[19] Milne I, Shaw P, Stephen G, Bayer M, Cardle L, Thomas WT, et al Flapjack—graphical genotype visualization. Bioinformatics. 2010;26(24):3133–4. 10.1093/bioinformatics/btq580 20956241 PMC2995120

[20] de Bourcy CF, De Vlaminck I, Kanbar JN, Wang J, Gawad C, Quake SR. A quantitative comparison of single-cell whole genome amplification methods. Plos One. 2014;9(8):e105585 10.1371/journal.pone.0105585 25136831 PMC4138190

[21] Kunzel G, Korzun L, Meister A. Cytologically integrated physical restriction fragment length polymorphism maps for the barley genome based on translocation breakpoints. Genetics. 2000;154(1):397–412. 10628998 10.1093/genetics/154.1.397 PMC1460903

[22] Sayed H, Kayyal H, Ramsey L, Ceccarelli S, Baum M. Segregation distortion in doubled haploid lines of barley (Hordeum vulgare L.) detected by simple sequence repeat (SSR) markers. Euphytica. 2002;125(2):265–72. 10.1023/A:1015861610226 .

