# Supplementary figures and images for: Measuring Meiotic Crossovers via Multi-Locus Genotyping of Single Pollen Grains in Barley
Source: PLoS One. 2015 Sep 10;10(9):e0137677. doi: 10.1371/journal.pone.0137677 (PMC4565660; doi:10.1371/journal.pone.0137677)

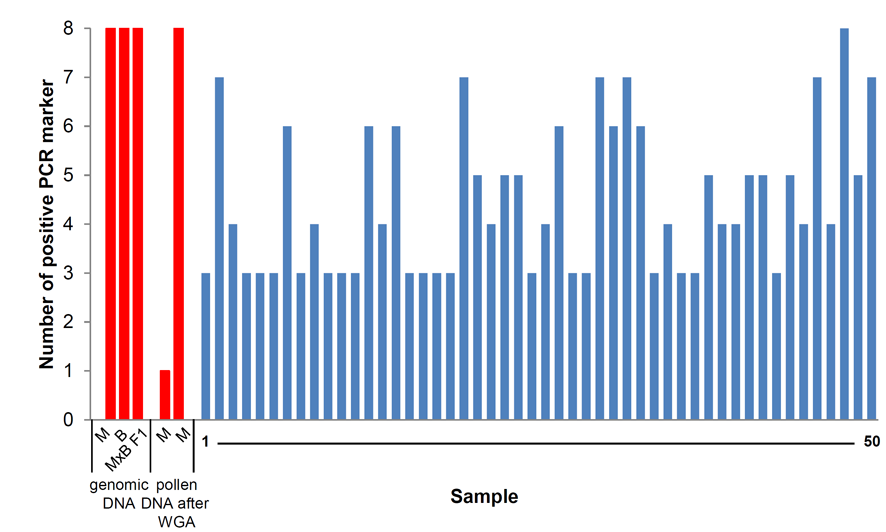

Supplement: S1 Fig — (TIF) [file pone.0137677.s001.tif]

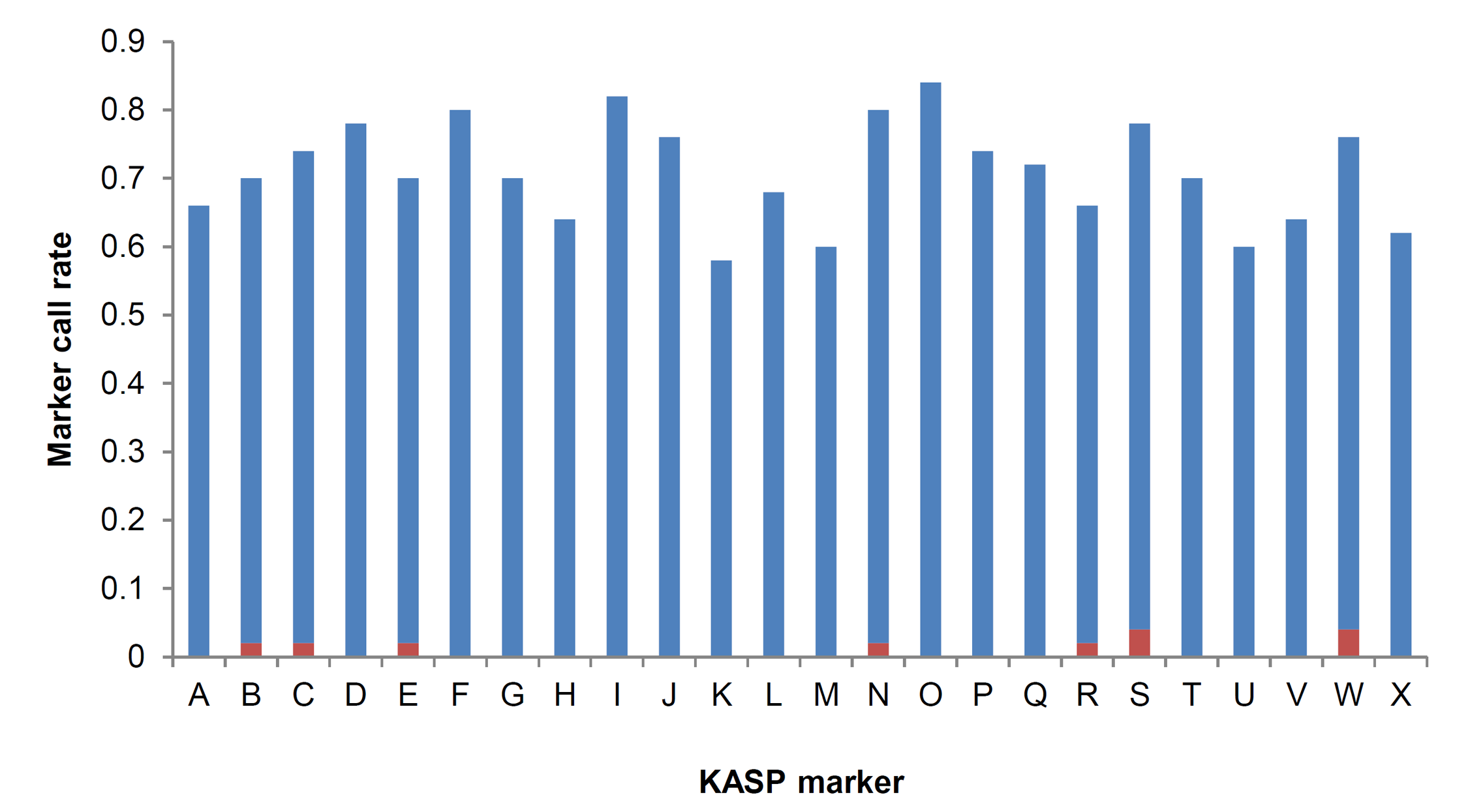

Supplement: S2 Fig — The marker call rate of each KASP marker (A to X) is shown (blue) as well as the frequency of presumptive double crossover (red), e.g. a crossover on both sides of a given marker. (TIF) [file pone.0137677.s002.tif]
