# Supplementary material for: Measuring Meiotic Crossovers via Multi-Locus Genotyping of Single Pollen Grains in Barley
Source: PLoS One. 2015 Sep 10;10(9):e0137677. doi: 10.1371/journal.pone.0137677 (PMC4565660; doi:10.1371/journal.pone.0137677)
Supplement: S1 Table — (DOCX) [file pone.0137677.s003.docx]

**Suppl. Table 1. List of primers used to evaluate WGA performance**

| **Primer name** | **Sequence 5' - 3'** | **Product size (bp)** |
| --- | --- | --- |
| P1-1F | GCAGCTTCACCTCATGTTCA | 100 |
| P1-1R | GCAGACCAACCTGACCCTAC |  |
| P13-1F | GGCACTCGCTCACATTCTTA | 87 |
| P13-1R | GGGAAGGCATGAACAACACT |  |
| P14-1F | GAAAGCAGACGCAGACACAG | 92 |
| P14-1R | AGCAGCAGCAGCAACTAATG |  |
| P15-1F | TGCTTCGTATGTGGACTGAACT | 96 |
| P15-1R | GCAACATCCCTGTCATCAATAA |  |
| P4-1F | CGATATGGCAGATGGGGTAT | 109 |
| P4-1R | CAGATGGCTGGACTTTGACA |  |
| P6-1F | CCTCGTCCTCCAATCAGTCT | 136 |
| P6-1R | CACCCGAACCCTAGATGTGT |  |
| P7-1F | CCCACACTGCCATACTCTCA | 106 |
| P7-1R | CCTTTTTGCAGCGTTTTCTC |  |
| P8-2F | AGAAGAACTCGCCGTACAAGAT | 117 |
| P8-2R | GAGGACCCCTTAATCCAGAACT |  |
| cereba2-F | CACATGGATACAACCGCAAC | 114 |
| cereba2-R | CAGGAACATAGCGACCATCA |  |
