# Supplementary material for: Measuring Meiotic Crossovers via Multi-Locus Genotyping of Single Pollen Grains in Barley
Source: PLoS One. 2015 Sep 10;10(9):e0137677. doi: 10.1371/journal.pone.0137677 (PMC4565660; doi:10.1371/journal.pone.0137677)
Supplement: S2 Table — (DOCX) [file pone.0137677.s004.docx]

**Suppl. Table 2 Gene-based KASP markers**

| **KASP marker** | **Gene** | **Chromosome** | **Physical map position (Mbp)** | **Polymorphism [Morex/Barke]** |
| --- | --- | --- | --- | --- |
| A | AK252051.1 | 3H | 2.94996 | TAACCACCAGCAAGCAAACAAGCCCTCTGTTTGCATGAGTGCGCAATATAACTTACATCAAGAAACCCCACAAACTGAGGAAC**[A/G]**AACATCATTTTGTACAGAGACAGCACATGCCACTCTCACATCTGTGATAAATATGCAAAGTGAGATGAAATAATTAGCACACAAAAACCCTTGTGACTGACCTCACCCATGGCACCCCGT |
| B | MLOC_62156.1 | 3H | 4.50244 | AGATAACCTMAAATCAGCTCTACAACAATTTGGTGCCCTGGAGGGCTAGAATCAATCACC**[A/G]**ACTTGTCACAGYCGGCAGCGGAATAAAGTAGGTGTGTGGATGCACACTTTGCTATGGGGT |
| C | MLOC_36532.1 | 3H | 8.01784 | GCCGCCAGAATGATAGTATGCCGACGGAGGAGCTTGTGGAAACTCAGGTGGAAGGTAGAAGTCAAAGAAGAATAGGCCATCTTGGTATGGTGTGCCACTTGCTCCAATAATCACAGCTCT**[A/C]**ATGAGATCCATACGATCTTCAAATACCCTCACATAAATATAATCTGGTAGGTTCTTCTCAAGTATGCCCCATTCTTGCTGAACTTTTTTTACCCACTTCCTTCCACCGGTACCATGAGCT |
| D | AK372389 | 3H | 13.6276 | CGGGAGAATGAACAGAGCAATCACGTGCGATATGGCTTGCAGAATACCGTCCAATGGATGGAATGCTAGTCCAGCAAATGGTGATAGGGTATTCTCCTTGTTGTAAATGTGGTGGGTTGC**[A/G]**TGTAGGTGCTTATACAGTGGTTTTATGTCATGCAACTCTCTGTGCATCCAGTAAATTCCAAACTCCACAAAGGTGAGATATAAAGCCACATATACAAAATACATCGGCCAACCAACTTCA |
| E | AK248960.1 | 3H | 47.14852 | GAAGGTAGAGGTCCCCCTGTCGAGGACCCACAGCGCAAACATGTTCTGCGACTGGCGCTTGACCCTTATGAGGTCGCCGGATGGAGTCTCCACCAGGCAATTGAACTTCTGGTGCTGRTA**[A/G]**TGGACGAGGTTGTGCATATGCGGTTCCACCTGGGGGAAATCCTCATCTGGCCATGAACGGGGAGGCTCTGCCCACACCTCCACCGTACCATATACATCAATGGTGTAAAGTTGCCTTCTG |
| F | AK250083.1 | 3H | 50.33532 | CCGCAGCCGCCGTGCCACCGGGATGACCCTCGCCTTCCTCACCGCTGCTGCTAACGAGCT**[C/G]**GGCTGCCCCCTTCCTTGCTGACAAGGCCATATTTGAAGCTGACAATCAGCAACACTTGAC |
| G | AK365312 | 3H | 89.0754 | GATATGAGGAGAGGATCAGGCTGGTCATGTTCAACACCCATTTTGGAGCCCCCCACAAGC**[A/G**]CATCCATCCAGAGTCTTTCTACACAAACTCATGGCCAGAGTGCTTCTGCCAGATGAATGC |
| H | AK359814 | 3H | 117.73428 | TACAACCGAGATCCTGCAAGCAGGATGCCTCTAGCTAGCACAACCGGATTATTGATCATCATACAGACATCCATAACTTAAGAAACTTTACAGAACCAAATATCAGCAGCTAATAATATA**[A/G]**TTAAGCGCAACCACGACGTTATTATTTTATACAGTAATAAAATACAAAAAAGGAACGCAGCGCACGAACTTAGCAAGAGCTACATGCATGTAGTAGAAGCAGAAACCCGACTTCTACTTC |
| I | MLOC_54754.1 | 3H | 349.5932 | ACTTCTCGATCTGGCTGTCCATGACGACCAGCGCCGAGGAGTCGCCGACGTAGTAGGGGTGGTTCCCTGACCAAACATCCACCGAGTACTCCGGCTTCGTGCCCCCCGTCACCAGCACCA**[A/G]**CTCACCGTTGCAGTACACCTTCGCGTCCTCGAARATCTCCGGGTGCAGCCCCTTCTTCCGCATCGAGCACCTCATCCTCTGGGAGGGGACGAGGGAGCGGAGGGTACGGTTTGAATTTCT |
| J | AK367893 | 3H | 407.21724 | CCATCAGAAGGGYGCATTGTTGTGGATGAAGTCTGCTGCTGTMTACCTGTCTCTGTACTT**[A/G]**TTGTTCTGTCTACGTTGTAAGCATATGTTGTTACGCCCGTTTCGAGCTGCTTGTGATGAT |
| K | MLOC_39198.3 | 3H | 407.95256 | TCCGACTGAGTATTTGGGAGTCAAATTGGTGTCTTTCGGCTTTGCTGGACAAGGAAGAGC**[A/C]**ATCATGCGAGGTCCAATGGTTTCAGGAGTCATTAATCAGTTGCTGACCACGACTGACTGG |
| L | AK252342.1 | 3H | 423.49472 | TGTTGCCTATGAAACATTTGGTTTAACCACAGGTTTCATGTCAAGGACACGGTGTATCCT**[A/C]**TCCTCTATGTAACATCCTATTAGTGTTCTAGTCTGGAATTTGTTAGCTTGAAGCATTCTT |
| M | MLOC_2480.1 | 3H | 429.59304 | AGAAACTCACAGGCAAGCTATTGTTTCTGCTGTAACGAGTGGGAGCAGACAATACTTTCT**[T/C]**GGTACCGACAGTGCTCCCCATGATAAACGGATGAAGGAGTGCTCCTGTGGATGTGCTGGA |
| N | MLOC_10890.2 | 3H | 441.08536 | GTCTTCAACATTGAGCATCCACGTGTTACCGGAAGACTGGTCGATGGTGGTGTTAGTATT**[A/G]**TCCCTACCATATTGTTCAGCCTCGCTTGCTCCTGAAGAACTCTCGGGGCCTTGATGGCTG |
| O | AK363304 | 3H | 445.6994 | CAGGTCTCATCTACCAAATTTGAAGTACCTCCTTCAATGATCCCAGACATGTAACCACTTTCAAATTTAGCCTTGTTGGCAAAATATTTCTAGTGAACCTTGTATATTCTGATTTCTGCC**[A/G]**AAAGAATTATGCATGAAGAAGCACAATTCTTAGTCTACAGCTTGTGGTTCTGCATTTGAATCTCAAAACTGCCTCAAAAGTTATGCAGAGAAGTCAAGTTCAGCTTCCACAAAGAGAAGA |
| P | AK357864 | 3H | 459.52416 | TCACAACTAATTGCTGAATTCACCAATCGTAATCTGGCTGGTATTTAACATGCTGTTGTA**[A/G]**AATAGTTGATCGCGACTTCACATGCTTCTGCGCCCGAAGACATGTATGTCACGGATGTGT |
| Q | AK355032 | 3H | 465.49724 | GTCGTTGCCCAGGAGCATGGCTATGGCGTTGTTGAAGACGGACGAGTCGGCCGTGTTCCC**[A/G]**GAGAAGAACGCGTCTGGCCTCAAGTTTTCAGGATGAGACGAAGTGTCCCCTGCCCCGGGC |
| R | AK251764.1 | 3H | 468.14092 | GCCATATATAATTCTGCACATTCCTTTCTGATATACAGGAGAGCTGGGAGTGAGACAATG[A/G]AATCGAAGATATCTACTGTGGTGTTTCTGATCTCAAGCATATAGTACACCCGATGGTCCT |
| S | AK365737 | 3H | 484.32664 | GCTACCAGGTCCTGCATATCTTGGGCTCAGCAAATCATAGTTCTGAGAATAAAGTGCACC[A/G]GTCTCGAATAAGTGTGGGCTTGTCCGCAACTTTTCTGAATCTGGTGAAAGTTTTTTTTTT |
| T | AK354719 | 3H | 484.62176 | GCGGGCTCTCCGGCGCTGGAGCGGGAGGAGCAGGCTATAAATAAGGGGGGTGGGGCGCCCACAAGACAGAACCACACACCACAAGCAAGAGTGCACAAAAGCATACTGACCGCAGAGACC[A/G]CAGTGTTGAGATAGGATGGCGTCGGAGATGAGCAAGGACGTGAAGTTCTCCGACGAGGAAGTCACCTCGCACCCGCGCGTCGGCGAAGGTGAGGAGCAAACGGTTGGGCCGGCGCGGCAG |
| U | MLOC_13043.1 | 3H | 487.0162 | TGGTGTCAGTTGGGTAGAGCTTAAGGGTCAAGTGCATCAGTTCTGCACAGGAGATAAATC[T/C]CATCCAAGGCAAGGAGAGATCGATGAGTACTTGGAAGAAATGATGGCCAAAATCAGGCAA |
| V | AK367638 | 3H | 492.51312 | CTTATTGACTGTAATGAGGGGTGTCCTCTGACTGTTTGAGGGTATGGAGAAACTGCGAGC[T/C]AAGGAAGCAGGAGGCCGCGGAAGTGGCGAGGCTGTCTGCGATGCTGTTGGTGAGGCACGG |
| W | AK370453 | 3H | 506.33044 | GCGTCGACGCTCATGTCGAACACTTCGTTTCTGTGCAGCTCAGGCGCAGTGTAGTAACCT[A/G]AATCACAGGAGAGTGCGATTAAGTATATGTCAGCGAAGCATTATCT |
| X | AK355583 | 3H | 534.45488 | GTGATGTCAAGACCATTATTTTTGCTAGTTTTTACATAGTACAGTAGCAAGGGAAACTTC[A/C]AATATGCAGCAGCTTCACCCAAGTGGGTTCTTCAGCGAATATATTTAGCCCAGGAATAGT |
